# Effects of Nursing Intervention Based on Health Belief Model on Self-Perceived Burden, Drug Compliance, and Quality of Life of Renal Transplant Recipients

**DOI:** 10.1155/2022/3001780

**Published:** 2022-05-05

**Authors:** Shuqin Hu, Rui Xiong, Qingxiang Hu, Qingling Li

**Affiliations:** ^1^Organ Procurement Organizations, Jiangxi Provincial People's Hospital, Nanchang 330006, Jiangxi Province, China; ^2^Department of Organ Transplantation, Jiangxi Provincial People's Hospital, Nanchang 330006, Jiangxi Province, China; ^3^Outpatient Department, Jiangxi Provincial People's Hospital, Nanchang 330006, Jiangxi Province, China

## Abstract

**Objective:**

To explore the effects of nursing intervention based on health belief model (HBM) on self-perceived burden, drug compliance, and quality of life of renal transplant recipients.

**Methods:**

Sixty patients with renal transplantation treated in our hospital from February 2019 to July 2021 were enrolled. The patients were randomly assigned to control group and study group. The former received routine nursing and the latter received nursing intervention based on HBM.

**Results:**

The nursing satisfaction in the study group was higher compared to the control group (*P* < 0.05). Secondly, we compared the scores of self-burdens. Before nursing, they exhibited no significant difference (*P* > 0.05); after nursing, they decreased. Moreover, the physical burden, economic burden, and emotional burden of the study group were lower compared to the control group (*P* < 0.05). In terms of drug compliance, the rates of no missed medication, noncontinuous missed medication, timely medication, dose-by-dose medication, and non-self-stopping medication in the study group were higher compared to the control group (*P* < 0.05). The scores of SAS and SDS exhibited no significant difference before nursing (*P* > 0.05). After nursing, they decreased. Furthermore, the scores of SAS and SDS of the study group were lower compared to the control group (*P* < 0.05). The self-management ability exhibited no significant difference before nursing (*P* > 0.05); after nursing, it increased. Moreover, the self-management ability of the study group at discharge and 1 month, 3 months, and 6 months after discharge was higher compared to the control group (*P* < 0.05). Finally, we compared the scores of quality of life. Before nursing, there was no significant difference (*P* > 0.05). The scores of physiological function, psychological function, social function, and health self-cognition in the study group were lower compared to the control group (*P* < 0.05).

**Conclusion:**

The nursing intervention based on HBM can enhance the medication compliance of renal transplant recipients, and the intervention effect is long-lasting. Meanwhile, it can effectively enhance the negative emotion of patients, reduce the burden of self-feeling, promote the quality of life, strengthen the self-management of patients, and facilitate the prognosis.

## 1. Introduction 

The increased incidence of end-stage renal disease (ESRD) has significantly increased patients' demand for kidney transplantation [1]. The inevitable choices of ESRD are hemodialysis, peritoneal dialysis, and renal transplantation. Regarding dialysis, transplant patients have longer survival time, higher quality of life, and lower economic burden. At present, renal transplantation is the most effective method for the treatment of all kinds of ESRD, with the earliest clinical development, the largest number of transplantation cases, and the most mature parenchyma organ transplantation [2]. According to the results of the World Health Organization (WHO), there were 90306 kidney transplants, 32990 living donor kidney transplants (36.5%) and 57316 cadaveric donor kidney transplants (63.5%), in 2017 [3]. According to the US Organ Acquisition and Transplant Network, a total of 20119 kidney transplants were performed in the United States in 2017, including 5811 living donor kidney transplants (28.9%) and 14038 cadaveric donor kidney transplants (71.1%). In 1960, the first cadaveric kidney transplantation in China was successfully carried out by Academician Wu Jieping, a famous urologist [4]. At present, kidney transplantation in China is mainly organ donation after the death of citizens. According to the Chinese kidney transplantation scientific registration system, a total of 10793 kidney transplants were completed in 2017, of which 1753 were living donor kidneys, accounting for 16.2% of the national kidney transplantation. There were 9040 cadaveric donor kidney cases, accounting for 83.8% of the national kidney transplantation. From January to June 2018, 5873 kidney transplants have been carried out nationwide, including living donor kidney transplantation in 784 cases (13.3%) and cadaveric donor kidney transplantation in 5089 cases (86.7%). Since 1989, the short-term survival rate of renal transplantation has been greatly enhanced, but the long-term survival rate has been slowly strengthened [5]. According to the 2017 US annual report, the survival rate of transplanted kidneys is more than 90% one year after transplant, while the survival rate of 10 years after transplant is about 50%, with the survival rate of cadaveric donors being 46.4% and that of living donors being 61.4% [6].

Health belief model (HBM) is a model that predicts the influence of personal belief on behavior change [7]. This model holds that belief can influence behavior, highlights the leading role of belief in behavior, and believes that individual decision-making behavior is greatly influenced by subjective psychology [7, 8]. HBM includes understanding of disease threat; self-efficacy; and prompting, influencing, and restricting factors. The cognition of disease threat refers to the individual's subjective cognition of disease and health, including the severity and susceptibility of the disease, and the effectiveness of prevention and obstacles in action. The perception of disease susceptibility is the probability of the occurrence of the disease; the perception of the severity of the disease is the individual's understanding of the serious consequences of the disease; the perception of the benefits of healthy behavior is the individual's knowledge that it is good for their health to complete a certain behavior. The cognition of behavioral disorder is the individual's cognition of the obstacles and problems that may need to be faced to complete a certain behavior, including physical, psychological, time-related, economic, and other difficulties [8]. Self-efficacy refers to the individual's ability to complete a behavior and achieve the expected results in a specified situation, and it is the individual's own judgment on his or her own related abilities. The cue factors are the factors that promote the completion of a certain behavior, such as the promotion of manuals and books, and the illness of colleagues, relatives, or friends. The influencing and restricting factors include uncontrollable external factors such as age, sex, nationality, character, and educational level. HBM is widely employed in health education, such as explaining the transformation and maintenance of various healthy behaviors, or forming an important theoretical framework to guide behavior intervention. HBM is applied through the analysis of individual health beliefs; the use of pictorials, brochures, and WeChat education platform; and other ways to promote the relevant content of healthy behavior, give individuals correct cognition to establish a good health belief, make them take the initiative to complete healthy behavior, and finally achieve the goal of preventing adverse consequences [9]. Based on this, the current study focuses on the effects of nursing intervention based on HBM on self-perceived burden, drug compliance, and quality of life of renal transplant recipients.

## 2. Patients and Methods

### 2.1. General Information

Sixty patients with renal transplantation treated in our hospital from February 2019 to July 2021 were enrolled. The patients were randomly assigned to control group and study group. The former received routine nursing and the latter received nursing intervention based on HBM. In the control group, the age was 30–62 years old, with an average of 45.91 ± 3.63 years, including 18 males and 12 females, while in the study group, the age was 31–65 years old, with an average of 45.96 ± 3.58 years, including 16 males and 14 females. There was no statistical significance in the general data of the two groups. This study was permitted by the medical ethics association of our hospital, and all patients signed informed consent.

The inclusion criteria were as follows: (1) age > 18 years old; (2) transplantation time ≥3 months; (3) graft function without regular dialysis; (4) poor compliance with immunosuppressive drugs; (5) postoperative calcium neuroprotein inhibitor being tacrolimus; (6) recipients having no serious infection and complications after operation; (7) clear consciousness and ability to communicate in speech or writing; and (8) informed consent to participate in this study.

The exclusion criteria were as follows: (1) recipients of combined multiple-organ transplantation; (2) recipients of secondary or multiple renal transplants; (3) patients with severe organic diseases of heart, brain, lung, and other important organs; and (4) those who had participated in other clinical trials.

The shedding criteria are as follows: (1) after being informed of the interview time, the patients did not participate in any interview; (2) after the first interview, the patients were not present for three consecutive interviews. Patients with the above conditions were treated as shedding, and they were automatically withdrawn from the study.

### 2.2. Treatment Methods

The control group received routine nursing intervention in the department, the disease guidance manual was issued to the patients on the day of admission, the admission was evaluated, health education was patiently provided for the patients, and the matters needing attention in disease-related self-management were explained to them. Individualized nursing guidance was given, and the way of education was mainly through health education.

On the basis of the control group, the study group carried out nursing intervention based on HBM, and the specific measures were as follows: (1) In the first month of the intervention, provide the patients with the drug-taking manual for renal transplant recipients, focusing on the benefits of transplantation, the necessity of taking immunosuppressive drugs, the consequences of taking immunosuppressive drugs, the taking methods, and the matters needing attention regarding various immunosuppressive drugs. Fill in the medication plan and schedule according to the personal conditions of the patients. Ask the patient to fill in the medication schedule for the next month, focusing on the circumstances under which the medication noncompliance behavior is triggered (such as forgetting and not reminding the family), the status at this time (leisure, busy), and the behavior of drug noncompliance (such as missing or mistaking), used to judge the situation and causes of the patient's drug noncompliance. Sign the behavior agreement, the patient self-guarantee that he/she will take the medicine in accordance with the doctor's advice. (2) In the second month of intervention, focus on the methods of blood concentration monitoring and matters needing attention to keep the blood concentration stable, the consequences of rejection (a small amount), and the occurrence and treatment of infection (overdose) after taking immunosuppressive drugs without the doctor's advice. Determine the medication disorders of patients according to the immunosuppressant treatment disorder scale and the guidelines for intervention measures for common drug compliance disorders, and provide measures to solve the disorders according to the recommendations of the guidelines and the patients and their families, such as providing medicine kits, setting alarm clocks, and family reminders. According to the behavior feedback results of the first month, adjust the medication schedule with the patients and their families, affirm and encourage the patients' positive change behavior, and put forward correction and advice about their negative behavior. (3) In the third month of intervention, focus on the prevention and treatment of complications, including matters needing attention in self-protection and lifestyle, as well as the introduction of self-monitoring indicators; guide patients to fill in daily records; according to the results of behavior feedback in the second month, adjust the medication schedule with patients, and give encouragement or advice about patients' behavior changes. (4) Follow up the patients by phone/WeChat/SMS semimonthly, ask the patients whether they have the knowledge of immunosuppressive drugs, answer their questions, and ask the patients if they have the occurrence and causes of noncompliance with immunosuppressive drugs. Provide appropriate treatment measures.

### 2.3. Observation Index

#### 2.3.1. Satisfaction

After consulting the literature and experts' discussion, we designed patients' follow-up satisfaction, with a total of 10 items, and recorded patients' satisfaction with follow-up management mode, health education, medical and nursing service, and appointment registration process [10]. It is assigned to four dimensions: very satisfied, satisfied, general, and dissatisfied. Satisfaction rate = “very satisfied” rate + “satisfied” rate + “general” rate.

#### 2.3.2. Self-Perceived Burden Scale (SPBS)

The SPBS, which was developed by Pedroso-Chaparro et al. in 2003, was employed to measure the SPBS score of patients with chronic diseases [11]. The Chinese version of the scale was employed in this study, and Cronbach's *α* coefficient was 0.910. There are three dimensions: physical burden, economic burden, and emotional burden. The lower the score, the lighter the self-feeling burden.

#### 2.3.3. Medication Compliance

In this study, Basel assessment scale was employed to measure the medication compliance of renal transplant recipients [12]. There were 6 items in the scale, namely, 4 negative score items, 1 two-classification option item, and 1 self-score item. The first four items (1a, 1b, 2, 3) were scored from “none” to “almost every day” or “more than 4 times.” The compliance of transplant recipients in the past 4 weeks was measured from four aspects: missing medication, continuous missing medication, taking medicine on time, and taking medicine according to dose. The total score of the first four items is 4–24, and the higher the score is, the worse the recipient's compliance with medication is. Cronbach's *α* coefficient was 0.697.

#### 2.3.4. SAS and SDS Scoring

As for the SAS score, the anxiety self-rating scale, compiled by Naif et al. [13], has become one of the most commonly employed psychological measurement tools for psychological counselors, psychiatrists, and psychiatrists. The higher the score, the more serious the anxiety symptoms. The total score of anxiety was lower than 50 as normal, 50–60 as mild, 61–70 as moderate, and more than 70 as severe anxiety. The number of negative items indicates how many items the subjects did not respond to, and the number of positive items indicates how many items the subjects responded to. With respect to the total rough score, the scores of 20 items are added together, and the demarcation is assigned 40 points.

Regarding the SDS score, self-rating depression scale (SDS), compiled by W. K. Zung in 1965, is one of the scales recommended by the US Department of Education, Health and Welfare for psychopharmacology research [14]. The cut-off value of SDS standard score was 53. 53–62 was mild depression, 63–72 was moderate depression, and more than 73 was severe depression.

#### 2.3.5. Self-Management Ability

The self-management ability was investigated with self-made questionnaire [15]. The scale included regular review, reasonable diet, taking medicine on time according to doctor's advice, sleep, and exercise. The total score was 100 points. The higher the score, the higher the self-management ability.

#### 2.3.6. Quality of Life Scale

The quality of life scale includes four subscales, namely, physical, psychological, social, and health self-awareness, with a total of 29 items [16]. Cronbach's *α* coefficient of the scale is 0.79 to 0.91. The scale was scored 1–5 grades. The lower the score, the higher the satisfaction.

### 2.4. Statistical Analysis

Using SPSS 21.0 statistical software, before statistical analysis, the measurement data were tested by normal distribution and variance homogeneity analysis to meet the requirements of normal distribution or approximate normal distribution, expressed as $\bar{x}$ ±*s*, and repeated measurement data were analyzed by repeated measurement analysis of variance. *T*-test was employed to compare the two groups, *n* (%) was employed as an example to represent the counting data, and *χ*^2^ test was employed to indicate that the statistically significant difference (*P* < 0.05).

## 3. Results

### 3.1. Comparison of Nursing Satisfaction

**Figure 1:**
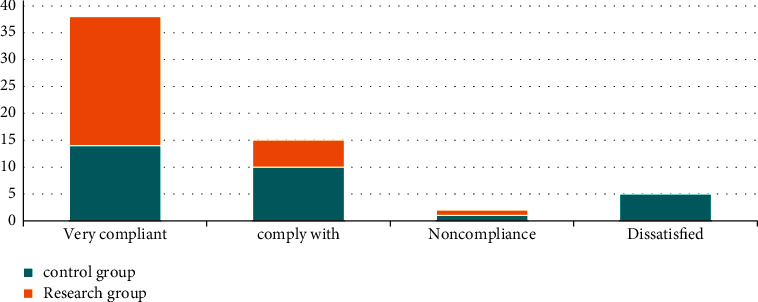
Comparison of nursing satisfaction between the two groups.

Comparing the nursing satisfaction, we found that the study group was very satisfied in 24 cases, satisfied in 5 cases, and general in 1 case, with a satisfaction rate of 100.00%; the control group was very satisfied in 14 cases, satisfied in 10 cases, general in 1 case, and dissatisfied in 5 cases. The satisfaction rate was 83.33%. Moreover, the nursing satisfaction in the study group was higher compared to the control group (*P* < 0.05). All the data results are indicated in Figure 1.

### 3.2. Comparison of Self-Burden Score

With regard to the scores of self-burdens, before nursing, there was no significant difference (*P* > 0.05). The physical burden, economic burden, and emotional burden of the study group were lower compared to the control group (*P* < 0.01). All the data results are indicated in Table 1.

### 3.3. Comparison of Drug Compliance

**Figure 2:**
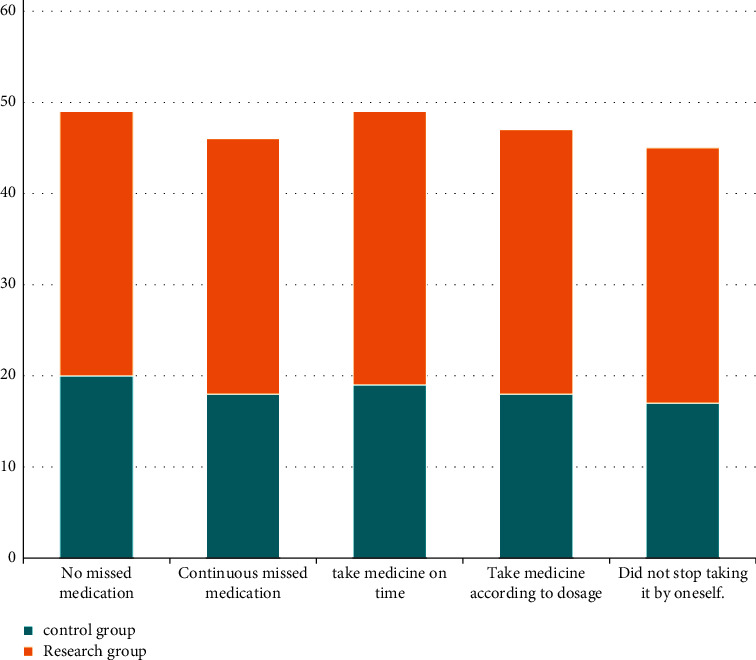
Comparison of medication compliance between the two groups.

Concerning the drug compliance, the rates of unmissed medication, noncontinuous missed medication, timely medication, dose-by-dose medication, and non-self-stopping medication in the study group were higher compared to the control group (*P* < 0.05). All the data results are indicated in Figure 2.

### 3.4. SAS and SDS Score Comparison

The scores of SAS and SDS exhibited no significant difference (*P* > 0.05) before nursing; after nursing, they decreased. Furthermore, the scores of SAS and SDS of the study group were lower compared to the control group (*P* < 0.05). All the data results are indicated in Table 2.

### 3.5. Comparison of Self-Management Ability

The self-management ability exhibited no significant difference (*P* > 0.05) before nursing; after nursing, it increased. In addition, the self-management ability of the study group at discharge and 1 month, 3 months, and 6 months after discharge was higher compared to the control group (*P* < 0.05). All the data results are indicated in Table 3.

### 3.6. Comparison of Quality of Life Scores

Before nursing, the scores of quality of life exhibited no significant difference (*P* > 0.05); after nursing, they decreased. Moreover, the scores of physiological function, psychological function, social function, and health self-cognition in the study group were lower compared to the control group (*P* < 0.05). All the data results are indicated in Table 4.

## 4. Discussion

ESRD is one of the important diseases that threaten the safety of human life, and its morbidity and mortality are relatively high. With the maturity of organ transplantation technology, renal transplantation plays a great role in saving patients' lives and improving the quality of life [16]. However, long-term medication is still needed after operation, and the probability of complications is also very high, so it is particularly important to provide continuous care for patients discharged from renal transplantation. It is necessary to take effective measures to strengthen nursing intervention and improve the sense of self-efficacy [17]. HBM is the earliest theoretical model adopted in the interpretation of individual health behavior. At present, it is widely adopted in the interpretation, prediction, and intervention of health behavior. The model was proposed by the American psychologist Rosenstock in 1966 and applied to the field of public health to explain why some people refuse to perform certain health-friendly behaviors, including perception of disease susceptibility and severity, perceived benefits and obstacles of healthy behavior, and cues [18]. As proposed by Janz and Becker, in this model, perceived behavioral benefits and barriers are subtracted from each other and directly affect behavior [19]. It is suggested that future studies should focus on more complex causal relationships such as health motivation and study the interaction between variables [20]. The HBM is mainly composed of three parts: personal perception, corrective factors, and possibility of behavior [20, 21]. The main results are as follows: (1) Personal perception includes perception of disease susceptibility and severity of the disease. When individuals realize the susceptibility and severity of the disease, that is, when they perceive the threat of the disease to themselves, they urge people to adopt healthy behavior or conduct disease screening. (2) Corrective factors refer to the factors that influence and modify an individual's perception of disease, including demographic variables, such as age, gender, and race; sociopsychological variables, such as personality, social status, and pressure from colleagues or groups; and structural variables, such as personal disease knowledge and disease experience. Prompt factors, such as individual's own symptoms of discomfort, publicity in the mass media, advice from relatives and friends, reminders from medical staff, and family or friends suffering from the disease can affect an individual's perception of the threat of disease. (3) The possibility of behavior includes perceiving the benefits of healthy behavior and perceiving the barriers to performing healthy behavior and self-efficacy. When individuals perceive the more benefits and fewer obstacles of adopting healthy behaviors and have the confidence to complete healthy behaviors, they are more likely to adopt healthy behaviors [22].

In terms of compliance, some scholars have discussed women's compliance with breast imaging examination based on HBM [23]. The results showed that the less perception of the benefits of breast cancer screening, perceptual disorders, and family history of breast cancer were important factors affecting compliance [23, 24]. In the studies of other scholars, based on this model, the influencing factors in medication compliance in patients with schizophrenia were comprehensively analyzed, and it was pointed out that individualized evaluation and treatment were the best [24]. By exploring the influencing factors in patients' compliance with antihypertensive drugs, some scholars point out that self-efficacy is the most important factor, and patients' compliance can be enhanced by strengthening self-efficacy [25]. Some scholars have also studied the compliance with anticoagulants in patients with cardiac valve replacement, and the results demonstrate that self-efficacy and perceptual behavior disorders are significant influencing factors [26]. In the field of renal transplantation, in 2012, some scholars discussed the factors that may affect the drug compliance of renal transplant recipients based on HBM [27]. It is concluded that renal transplant recipients with higher perception of medication disorders worry more about the use of immunosuppressive drugs, those with low self-control and lower life satisfaction are more likely to disobey instructions, and the most common reason for drug noncompliance is forgetting [27, 28]. The study pointed out that the medication disorders perceived by renal transplant recipients with poor compliance were mainly changes in daily living habits and lack of money. It is suggested that drug-taking behavior should be integrated into daily life and contingency plans should be made [28]. In 2017, some scholars pointed out that factors related to health beliefs such as perception of immunosuppressive drug disorders, long-term drug self-efficacy, drug treatment satisfaction, perceived drug knowledge, and social support were analyzed to explore the impact on drug compliance of renal transplant recipients [29]. The results showed that the main influencing factors of drug compliance of renal transplant recipients were drug taking disorder and social support. The main obstacles for patients to take drugs are the shape of the drug itself, the side effects of the drug, and the complexity of drug treatment. The more serious the patient's medication disorder is, the worse the medication compliance is [29, 30].

In terms of the intervention based on the HBM, some scholars have evaluated the effectiveness of the intervention measures in improving compliance guided by the HBM [30]. The study concluded that perceptual disorders and perceived benefits in the HBM are always powerful factors in predicting behavior. Some scholars have pointed out that the intervention measures based on behavior change theory can improve the compliance of the elderly [30, 31]. The results indicate that, for compliance intervention studies, there are differences in the use of different research theories and the effect of intervention. Han et al. have indicated that CD-ROM education and motivational telephone interviews can improve the blood pressure and medication compliance behavior of patients with diabetes and kidney disease, but the difference is not significant and needs to be further improved [31]. Gu et al. applied this model to implement long-distance health education through mobile client app, which significantly enhanced the compliance of breast cancer patients with endocrine therapy [32]. Zhao et al. apply health education based on HBM to improve drug compliance of pulmonary *tuberculosis* patients [33]. Most of the intervention studies based on HBM are in the form of health education, which is a single-dimensional intervention, and there is no intervention research on drug compliance of renal transplant recipients based on HBM in China. In terms of the results of this study, nursing intervention based on HBM is patient-centered, focusing on stimulating patients' internal potential, arousing their enthusiasm and initiative, changing their behavior and consciousness, and improving the clinical outcome of renal transplant recipients. The results indicated that the scores of self-perceived burden, SDS, and SAS in the study group were significantly lower compared to the control group after intervention (*P* < 0.05), which fully shows that the nursing model based on HBM is suitable for the nursing process of patients after renal transplantation and helps to form a good cooperative relationship between nurses and patients. Nursing intervention based on HBM can stimulate patients' motivation to change their behavior and urge them to assume the responsibility of self-management by clarifying problems, making steps, and implementing plans after communicating with patients. In this model, nurses have changed their leading role and put more emphasis on helping patients learn all aspects of relevant knowledge and distinguish the pros and cons of their behavior. Providing patients with problem-solving solutions directly is not advocated, which can effectively enhance the self-management ability of patients and be more scientific. This study indicated that the scores of physical function, social function, role function, and cognitive function in the study group were higher compared to the control group, and the drug compliance in the study group was significantly higher compared to the control group (*P* < 0.05). This indicates that the nursing intervention based on HBM can help patients effectively restore the function of renal transplantation and promote the quality of life. The reason may be that according to the guidance of HBM, this study implements comprehensive nursing intervention of health education, behavior change, and emotional intervention, while providing knowledge of drugs and diseases to renal transplant recipients, paying attention to solving their medication disorders, holding monthly face-to-face interviews to mobilize their subjective initiative to a certain extent, listening to their medication experience, clearing up their confusion, giving them emotional support, and encouraging them to actively manage medication behavior.

Conclusively, the nursing intervention based on HBM can enhance the medication compliance of renal transplant recipients, and the intervention effect is durable. Meanwhile, it can effectively strengthen the negative emotion of patients, reduce the burden of self-feeling, enhance the quality of life, facilitate the self-management of patients, and promote the prognosis.

## Figures and Tables

**Table 1:** Comparison of the scores of self-burden between the two groups [$\bar{x}$ ± *s*, points].

Group	*N*	Physical burden			Economic burden		Emotional burden		
		Before nursing	After nursing		Before nursing	After nursing	Before nursing		After nursing
C group	30	18.43 ± 3.55	15.49 ± 3.31^a^		16.59 ± 1.24	10.28 ± 1.22^a^	14.32 ± 1.33		11.56 ± 1.53^a^
R group	30	18.64 ± 3.41		11.21 ± 1.21^b^	16.54 ± 1.56	3.56 ± 0.66^b^	14.67 ± 1.24	7.43 ± 1.22^b^	
*t*		0.233		6.651	0.137	26.535	1.504	11.559	
*P*		＞0.05		＜0.01	＞0.05	＜0.01	＞0.05	＜0.01	

*Note.* Compared with the control group before and after nursing, ^a^*P* < 0.05; compared with the study group before and after nursing, ^b^*P* < 0.05.

**Table 2:** Comparison of SAS and SDS scores between the two groups [$\bar{x}$ ± *s*, points].

Group	N	SAS		SDS	
		Before nursing	After nursing	Before nursing	After nursing
C group	30	64.29 ± 3.66	54.93 ± 3.65^a^	76.59 ± 4.31	65.97 ± 4.35^a^
R group	30	64.34 ± 3.52	41.29 ± 3.31^b^	76.42 ± 4.67	43.19 ± 4.54^b^
*t*		0.053	15.162	0.146	19.843
*P*		＞0.05	＜0.01	＞0.05	＜0.01

*Note.* Compared with the control group before and after nursing, ^a^*P* < 0.05; compared with the study group before and after nursing, ^b^*P* < 0.05.

**Table 3:** Comparison of self-management ability between the two groups of patients [$\bar{x}$ ± *s*, points].

Group	*N*		Before nursing	When discharged from the hospital	1 month after discharge	3 months after discharge		6 months after discharge
C group	30		54.91 ± 3.31	60.49 ± 3.74	67.48 ± 4.75	76.59 ± 3.75	81.72 ± 3.66	
R group	30		54.69 ± 3.45	67.38 ± 3.55	74.92 ± 3.35	80.54 ± 4.12	89.49 ± 3.31	
*T*			0.252	7.318	7.010	3.883	8.624	
*P*			＜0.01	＜0.01	＜0.01	＜0.01		＜0.01

**Table 4:** Comparison of quality of life scores between the two groups before treatment [$\bar{x}$ ± *s*, points].

Group	*N*	Physiological function		Psychological function		Social function		Healthy self-cognition	
		Before nursing	After nursing	Before nursing	After nursing	Before nursing	After nursing	Before nursing	After nursing
C group	30	15.84 ± 4.64	13.66 ± 2.54a	16.12 ± 3.44	14.85 ± 4.86a	18.12 ± 3.66	16.55 ± 2.77a	15.42 ± 3.23	13.85 ± 1.33^a^
R group	30	15.13 ± 4.64	11.66 ± 2.67b	16.55 ± 3.53	12.81 ± 1.85b	18.23 ± 3.64	12.12 ± 3.77b	15.55 ± 3.33	10.13 ± 2.64^b^
*T*		0.592	2.972	0.477	2.148	0.116	5.186	0.153	6.892
*P*		＞0.05	＜0.01	＞0.05	＜0.01	＞0.05	＜0.01	＞0.05	＜0.01

*Note.* Compared with the control group before and after nursing, ^a^*P* < 0.05; compared with the study group before and after nursing, ^b^*P* < 0.05.

## Data Availability

No data were used to support this study.
